# Comment on “Association between frailty recovery and dietary variety among community-dwelling older Japanese adults”

**DOI:** 10.1016/j.jnha.2026.100853

**Published:** 2026-04-20

**Authors:** Funda Yildirim Borazan, Osman Talha Dogan

**Affiliations:** Gazi University, Faculty of Medicine, Department of Internal Medicine, Division of Geriatrics, Ankara, Turkey

Dear Editor,

We read with great interest the recent short communication by Hirose et al., examining the association between dietary variety and frailty recovery among community-dwelling older adults [1]. The authors reported that a higher dietary variety score was associated with a greater likelihood of recovery from frailty during a 1-year follow-up. These findings offer important insight into the potential role of nutrition in frailty trajectories.

However, in this study, greater dietary variety mainly reflected increased consumption of plant-based foods, especially soybean products. Soy foods are high in polyunsaturated fatty acids (PUFAs) [2] and contain bioactive components that may have anti-inflammatory effects and promote skeletal muscle health [3]. Therefore, the link between dietary variety and frailty recovery could, at least partially, be explained by these nutrient properties.

Similarly, in our previous study among older outpatients, we found that frail individuals had significantly lower intake of plant proteins, and PUFA intake was independently and inversely associated with frailty [4]. These results suggest that diet quality affecting frailty depends not only on diversity but also on the specific nutrient composition, especially plant protein and healthy fats. Furthermore, broader dietary patterns may also play a significant role in frailty outcomes [5]. For instance, a large cohort study found that adherence to a Mediterranean diet was associated with a lower risk of frailty in older women, supporting the idea that overall diet quality—not only individual food-group variety—can influence frailty risk [6].

Taken together, the findings of Hirose et al. and our study support the idea that both dietary variety and nutrient content can influence frailty outcomes. Emphasizing the role of plant-based proteins and PUFA levels may help explain the biological mechanisms behind the link between dietary variety and frailty recovery. Future studies integrating dietary diversity indices with detailed nutrient intake analyses could further inform targeted dietary strategies to prevent frailty and support recovery in older adults.

## Declaration of Generative AI and AI-assisted technologies in the writing process

Artificial intelligence–based tools were not used in the preparation of this manuscript.

## CRediT authorship contribution statement

**Funda Yildirim Borazan:** Conceptualization, Writing – original draft. **Osman Talha Dogan:** Conceptualization, Writing – review & editing.

## Declaration of competing interest

The authors declare no competing interests.
